# Notch dependent chromatin remodeling enables Gata3 binding and drives lineage specific CD8 ^+^ T cell function

**DOI:** 10.1111/imcb.70002

**Published:** 2025-02-26

**Authors:** Jessie O'Hara, Pushkar Dakle, Michelle Ly Thai Nguyen, Adele Barugahare, Taylah J Bennett, Vibha AV Udupa, Nicholas Murray, Gemma Schlegel, Constantine Kapouleas, Jasmine Li, Stephen J Turner, Brendan E Russ

**Affiliations:** ^1^ Department of Microbiology, Monash Biomedical Institute Monash University Clayton VIC Australia; ^2^ Department of Microbiology and Immunology, The Peter Doherty Institute for Infection and Immunity The University of Melbourne Parkville VIC Australia; ^3^ Monash Bioinformatics Platform, Monash Biomedical Discovery Institute Monash University Clayton VIC Australia

**Keywords:** CD8^+^ T cell, Gata3, Granzyme, influenza A virus, Notch, T cell memory

## Abstract

Activation of CD8^+^ T cells enable them to control virus infections and tumors. This process involves the differentiation of naïve CD8^+^ T cells into effector and memory states, driven by specific transcription factors (TFs). Previously, we have shown that Granzyme A (Gzma) induction in activated CD8^+^ T cells depends on Gata3 and the establishment of a permissive chromatin landscape at the *Gzma* locus. Interestingly, Gzma expression is independent of IL‐4 signaling, which typically upregulates Gata3 in CD4^+^ T cells, suggesting an alternative pathway for Gata3 induction. Here we demonstrate that Notch signals during CD8^+^ T cell activation promote Gzma expression. Inhibition of Notch signaling or loss of the Notch transactivator Rbp‐j leads to reduced Gzma expression, with transcriptionally repressive chromatin at the *Gzma* locus. The genome targets of Gata3 differ in effector CD8^+^ T cells activated with IL‐4 compared with those activated with Notch signals or isolated after IAV infection. This indicates that the signals received during CD8^+^ T cell activation can alter the chromatin landscape, affecting Gata3 function. Furthermore, Gata3 deficiency results in reduced IAV‐specific CD8^+^ T cell responses and decreased Gzma expression, although the *Gzma* locus maintains a permissive chromatin landscape. These findings suggest that Notch signals received by virus‐specific CD8^+^ T cells prepare the chromatin landscape for Gata3 binding to CD8^+^ lineage‐specific gene loci, promoting effective CD8^+^ T cell immunity.

## Graphical abstract

This study examines how Notch signaling conditions the genome landscape within activated CD8^+^ T cells in such a way to alter the binding pattern of transcription factors such as Gata3. This Notch dependent conditioning directs Gata3 binding to CD8^+^ T cell lineage specific gene loci enabling generation of effective T cell responses.

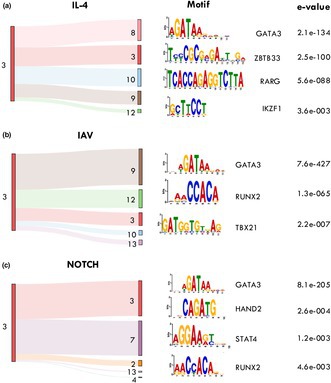

## INTRODUCTION

CD8^+^ T cells play an important role in the control of virus infection and tumors. Naïve CD8^+^ T cell activation results in a program of differentiation and proliferation that results in the generation of effector CD8^+^ T cells that exhibit cytolytic capacity via expression of granule enzymes (Gzm) and perforin (PRF),^1^, ^2^ as well as the ability to secrete pro‐inflammatory cytokines such as interferon‐γ (Ifn‐γ), tumor necrosis factor (TNF) and CCL5.^3^, ^4^, ^5^ Optimal naïve T cell activation requires the integration of multiple signaling cascades that include signals triggered by antigen‐specific T cell receptor ligation (Signal 1); co‐stimulation (Signal 2) and soluble factors such as cytokines (Signal 3).^6^, ^7^, ^8^ Once infection is controlled, the effector CD8^+^ T cell population contracts and establishes a long‐lived memory T cell population.^9^ In contrast to naïve CD8^+^ T cells, virus‐specific memory CD8^+^ can elicit immediate effector function, without the need for further differentiation.^10^ This contributes to an accelerated clearance of infection upon secondary challenge.

The changes associated with the naïve CD8^+^ T cell differentiation state into the effector and memory states correlate with engagement of distinct transcriptional programs and specific changes in the chromatin architecture.^11^, ^12^, ^13^, ^14^, ^15^ These molecular changes are driven by the stepwise induction of specific transcription factors whose coordinated expression are key for optimal CD8^+^ T cell differentiation. The transcription factors (TFs) Batf and Ifr4 are initially upregulated quickly after naïve CD8^+^ T cell activation and drive key aspects of the early differentiation program, including cell survival and metabolic gene transcription.^16^, ^17^ Subsequent upregulation of T‐bet, Eomes, Stat5 and Runx3 enable the acquisition of lineage specific effector CD8^+^ function and phenotype,^18^, ^19^, ^20^, ^21^ whilst expression of Zeb2 and Blimp‐1 finally commit activated CD8^+^ T cells to a terminal differentiation state.^22^, ^23^

Gata3 is a transcription factor typically considered to be a master regulator of activated CD4^+^ helper T cells (T_H_) commitment to the T_H_2 lineage.^24^, ^25^, ^26^ Activation of naïve CD4^+^ T cells in the presence of interleukin‐4 (IL‐4) upregulates Gata3 expression, which in turn plays a key role in the expression of IL‐4, IL‐5 and IL‐13, signature T_H_2 cytokines via chromatin remodeling.^27^, ^28^ Importantly, Gata3 has also been reported to have broader roles in a number of other immune cell subsets including being required for immature T cell development,^24^ regulatory T cell function^29^ and NK cell function.^30^, ^31^ In CD8^+^ T cells, Gata3 has been shown to be key for sustained CD8^+^ T cell proliferation upon activation,^32^, ^33^ in part via direct targeting of the cell cycle regulator c‐Myc.^33^ Moreover, Gata3 has been shown to have a role in ensuring optimal acquisition of CD8^+^ cytolytic effector function.^32^ We, and others, have demonstrated that *in vitro* activation of CD8^+^ T cells in the presence of Il‐4 signaling promotes Gzma expression in a Stat6 dependent manner.^34^, ^35^ IL‐4 dependent Gzma expression was associated with the establishment of a permissive chromatin landscape and Gata3 binding within the *Gzma* locus.^34^ Importantly, *ex vivo* isolated influenza A virus (IAV) specific effector CD8^+^ T cells also exhibited a permissive chromatin landscape and Gata3 binding at the *Gzma* locus.^34^ However, the establishment of this permissive chromatin structure and subsequent Gata3 binding at the *Gzma* locus in virus‐specific CD8^+^ T cells was found to be independent of Stat6, and hence, IL‐4 signaling.^33^ Together, this observation suggests that there is an alternative pathway that intersects with Gata3 binding to promote CD8^+^ T cell effector function.

The Notch family of cell surface receptors are key in regulating T cell development^36^ and in the generation of distinct CD4^+^ T cell lineages.^37^ T cell activation results in Notch1 and Notch2 cell surface expression where they ligate with Notch ligands of the Jagged and Delta‐like families expressed on dendritic cells.^37^, ^38^, ^39^ Notch signaling is mediated via enzymatic cleavage of Notch receptors that releases the Notch intracellular domain (Ncid). Ncid translocates to the cell nucleus where it interacts with the DNA binding protein Recombination Signal Binding Protein for Immunoglobulin Kappa J Region (Rbp‐j)^40^ and the co‐activator Mastermind‐like (Maml) resulting in gene transcription.^41^ Notch signaling has been shown to be an alternative pathway for Gata3 upregulation and T_H_2 CD4^+^ T cell commitment.^42^, ^43^ In CD8^+^ T cells, Notch signaling has been demonstrated to be a key factor in ensuring optimal effector CD8^+^ T cell differentiation and acquisition of function.^38^, ^39^ For example, Notch2 signaling results in Ncid forming a complex with CRE‐Binding protein 1 (Creb1) to activate Gzmb expression within *in vitro* activated CD8^+^ T cells.^39^ However, the precise mechanism of Notch action, and how this interacts with Gata3 to promote CD8^+^ effector T cell differentiation, is not fully understood.

Given that we have demonstrated that Gata3 upregulation and subsequent binding to the *Gzma* locus in virus‐specific CD8^+^ T cell was independent of IL‐4 signaling,^34^ we reasoned that the Notch signaling pathway may be an alternate pathway. Supporting earlier studies,^32^, ^33^ we demonstrate that T cell specific Gata3 deficiency results in diminished IAV‐specific CD8^+^ T cell effector and memory responses, with decreased cytokine and Gzma, but not Gzmb, expression. Mapping genome wide Gata3 binding in naïve and effector CD8^+^ T cells demonstrates that while there was some overlap in Gata3 genome targets, there were distinct Gata3 binding patterns in naïve and effector CD8^+^ T cells. Utilizing the OP9‐DL1 culture system, we demonstrate that genome wide Gata3 binding patterns induced by Notch signaling within *in vitro* activated CD8^+^ T cells best resemble that observed in *ex vivo* isolated influenza‐specific CD8^+^ T cells. Surprisingly, while Notch signaling promoted Gzma expression, this was not via direct upregulation of Gata3 expression. Subsequent chromatin analysis demonstrated that Notch signals result in chromatin remodeling of the *Gzma* locus into a permissive structure that then enables Gata3 binding. These data highlight the importance of extrinsic signals that help to shape the chromatin landscape in activated CD8^+^ T cells to ensure optimal acquisition of lineage‐specific function.

## RESULTS

### Integration of Notch signals upon activation promotes CD8 ^+^ T cell Gzma protein expression

**Figure 1:**
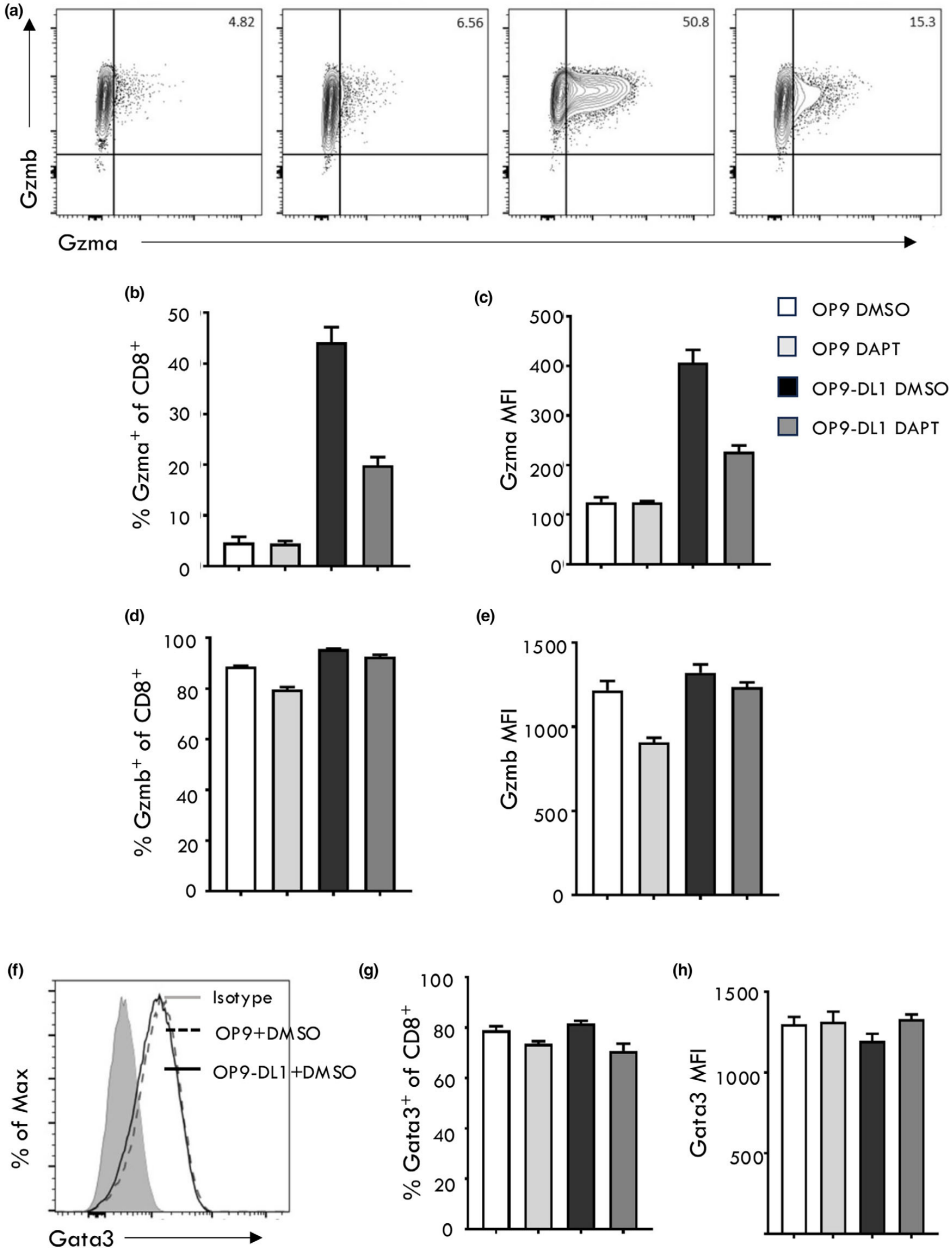
Notch signaling promotes Gzma expression in activated CD8^+^ T cells. Naive (CD44^lo^ CD62L^hi^) CD8^+^ OT‐I cells were sort purified and activated *in vitro* with anti‐CD3 and anti‐CD28 microbeads, in the presence of OP9 or OP9‐DL1 stromal cells as well as the gamma‐secretase inhibitor DAPT or the DMSO vehicle control. The cells were cultured for 4 days and levels of Gzma **(a–c)**, Gzmb **(a, d, e)** and Gata3 **(f–h)** were assessed by intracellular staining and flow cytometry. Data are shown as mean ± s.e.m. (*n* = 3) and are representative of three independent experiments.

While IL‐4 can result in Gata3 upregulation and promote Gzma expression after *in vitro* and *in vivo* activation of CD8^+^ T cells,^34^, ^35^ we have previously shown that Gzma expression by virus‐specific CD8^+^ T cells is independent of *in vivo* IL‐4 signaling.^34^ Given that Notch signaling has been reported to be an alternative signal for driving Gata3 upregulation,^42^ we reasoned that Notch signals may also promote Gzma expression. To initially test this hypothesis, we stimulated naïve (CD62L^hi^CD44^lo^) OT‐I transgenic CD8^+^ T cells *in vitro*, with polyclonal anti‐CD3/anti‐CD28 beads, in the presence or absence of Notch ligand signaling via the use of a OP9 cell line expressing the Notch ligand, Delta Ligand 1 (OP9‐DL1).^44^ Activation of OT‐Is in the presence of Notch signaling resulted in significant upregulation of Gzma expression (compare OP‐9 DMSO with OP9‐DL1 DMSO) (Figure 1a–c). In comparison, activation induced Gzmb expression was independent of Notch signaling (Figure 1d). The γ‐secretase inhibitor, DAPT (N‐[N‐(3,5‐difluorophenacetyl)‐l‐alanyl]‐s‐phenylglycine‐butyl ester), blocks the generation of the Notch Intracellular Cytoplasmic Domain (NICD) transactivator, and hence limits Notch signal transduction.^45^ The addition of DAPT to the OT‐I/OP9‐DL1 cultures resulted in a decrease in Gzma expression both in terms of proportion and MFI (Figure 1b, c). Interestingly, this was independent of Gata3 expression as the presence or absence of Notch signaling (including inhibition of NICD) did not significantly alter Gata3 levels (Figure 1f–h). These data suggest that while the promoter of Gzma expression is dependent on Notch signaling, it is not via direct upregulation Gata3 expression.

### Notch dependent chromatin remodeling enables Gata3 recruitment to the Gzma locus

**Figure 2:**
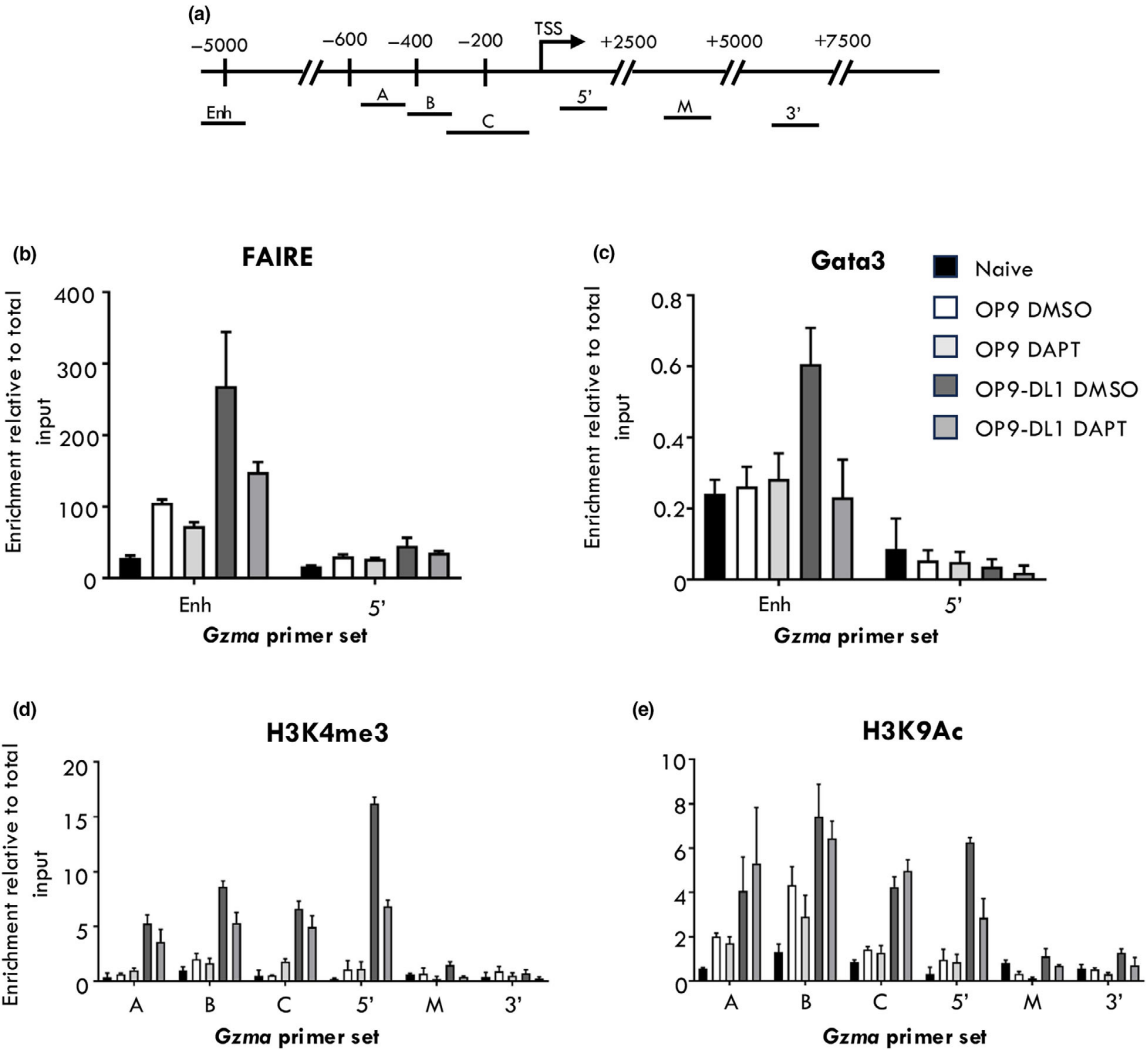
Notch signaling promotes a permissive chromatin landscape at the *Gzma* locus within activated CD8^+^ T cells enabling Gata3 binding. Naive (CD44^lo^ CD62L^hi^) CD8^+^ OT‐I cells were sort purified and activated *in vitro* in the presence of OP9 or OP9‐DL1 stromal cells as well as the gamma‐secretase inhibitor DAPT or the DMSO vehicle control. **(a)** Location of real‐time PCR using primer sets spanning the regulatory region upstream of the *Gzma* TSS (Enh), the *Gzma* promoter region **(a–c)** and the *Gzma* gene body (5′, M and 3′). Cells were cultured for 4 days and subjected to either FAIRE **(b)** or ChIP with antibodies directed against Gata3 **(c)** and the permissive histone marks H3K4me3 **(d)** and H3K9Ac **(e)**. Data are shown as mean ± s.e.m. (*n* = 3) of three independent experiments.

We have previously demonstrated that Gata3 binding to the Gzma locus is associated with chromatin remodeling to a transcriptional permissive state correlating with Gzma expression within virus‐specific CD8^+^ T cells.^34^ Given this was also demonstrated to be independent of IL‐4 signaling,^34^ we reasoned that Notch signaling could result in greater accessibility of the Gzma locus within recently activated CD8^+^ T cells enabling Gata3 binding. To test this, Gata3 ChIP was carried out on either naïve (CD44^lo^CD62L^hi^) or *in vitro* activated CD8^+^ T cells in the presence or absence of OP9‐DL1 cells as described previously above. Gata3 binding was observed at a previously reported non‐coding regulatory element located approximately 1Kb upstream of the *Gzma* TSS, but not at that *Gzma* promoter, when OT‐Is were activated in the presence of Notch signaling (Figure 2a). This correlated with an increase in chromatin accessibility (Figure 2b), and enrichment of transcriptionally permissive histone PTMs, H3K4me3 and H3K9Ac, particularly at the Gzma TSS (Figure 2c, d, respectively). Importantly, inhibition of Notch signaling decreased chromatin accessibility and Gata3 binding at the Gzma enhancer, whilst resulting in a decrease in permissive histone PTMs at the Gzma TSS (Figure 2a–d). Together, these data suggest that Notch signaling promotes chromatin remodeling of the *Gzma* locus, particularly at the *Gzma* enhancer, enabling Gata3 binding and the subsequent establishment of a permissive chromatin landscape across the *Gzma* TSS.

### Rbp‐j is a key mediator of Notch dependent chromatin remodeling at the *Gzma* locus

**Figure 3:**
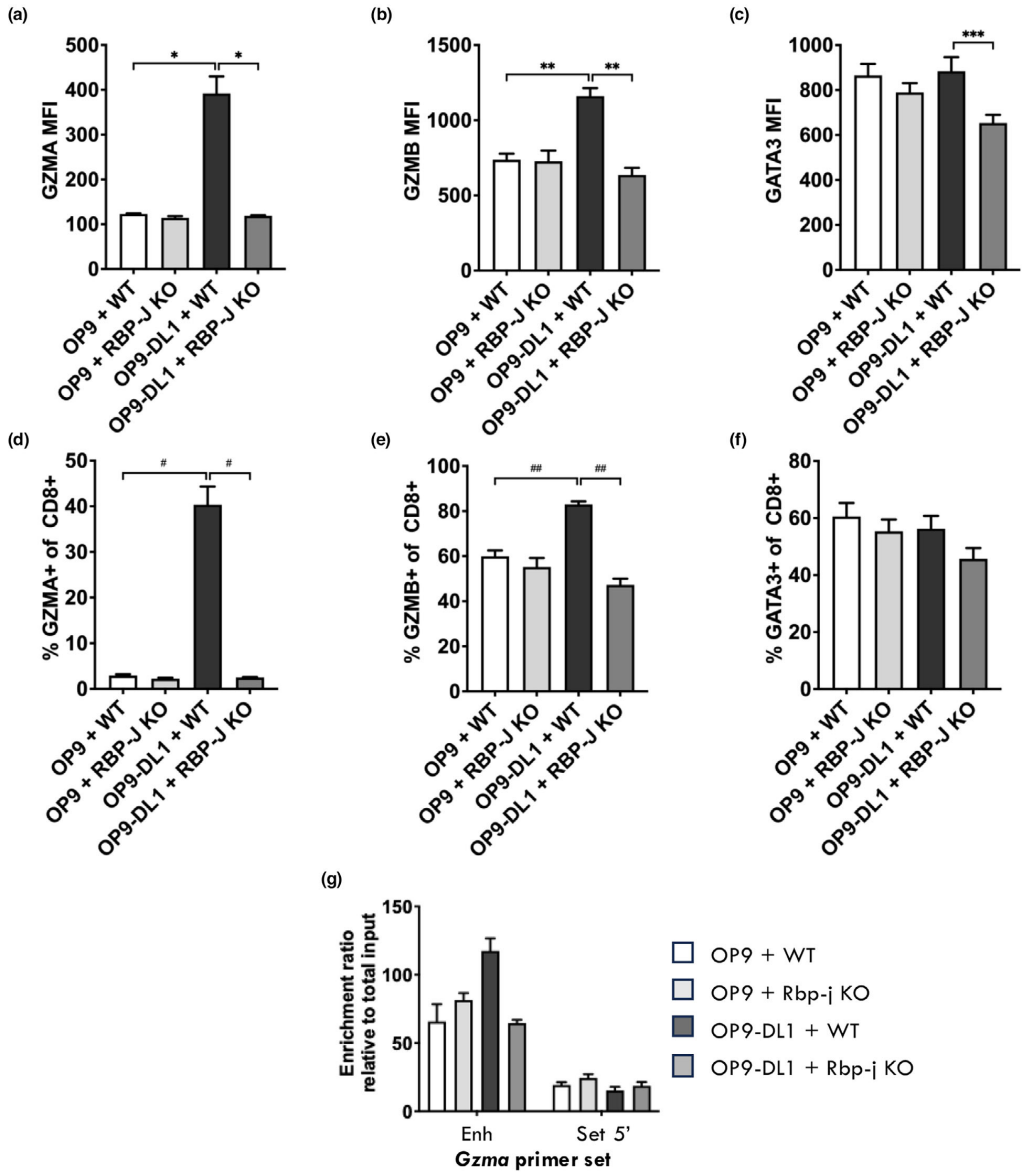
Notch dependent chromatin remodeling at the *Gzma* locus is dependent on the Rbp‐j transactivator and not Gata3. Naive (CD44^lo^ CD62L^hi^) wildtype (WT) or Rbp‐j deficient CD8^+^ OT‐I cells were sort purified and activated *in vitro* with 1 μg of SIINFEKL peptide, in the presence of OP9 or OP9‐DL1 stromal cells as well as the gamma‐secretase inhibitor DAPT or the DMSO vehicle control. The cells were cultured for 4 days and the expression level **(a–c)** and proportion **(d, e)** Gzma **(a, d)**, Gzmb **(b, e)** and Gata3 **(c, f)** were assessed by intracellular staining and flow cytometry. **(g)** Cells were cultured for 4 days and subjected to FAIRE and analyzed by real‐time PCR using primer sets spanning the regulatory region upstream of the *Gzma* TSS (Enh). Data are shown as mean ± s.e.m. (*n* = 3) of three independent experiments. *P < 0.01, **P < 0.02, ***P < 0.03; ^#^ *P* < 0.002, ^##^ *P* < 0.03; Kruskal‐Wallis non‐parametric test.

Notch activation results in NICD nuclear translocation where it pairs with a chromatin binding protein, Rbp‐j, to mediate transcriptional regulation.^40^ To determine the role of Rbp‐j in regulating Gzma expression within activated CD8^+^ T cells, Rbp‐j‐deficient OT‐I CD8^+^ T cells were activated *in vitro* with the ovalbumin peptide antigen (OVA_257‐264_; SIINFEKL, amino acid sequence) and anti‐CD28 agonist antibody, with and without Notch signaling, as described above. Rbp‐j deficient CD8^+^ T cells failed to upregulate Gzma in the presence of OP9‐DL1 cells (Notch signaling), there was some impact of Rbp‐j deficiency on Gzmb upregulation, while Gata3 expression was unaffected (Figure 3a–f). The fact that NOTCH signaling did appear to moderately boost Gzmb expression in this instance is at odds with Figure 1, but may be due to the different activation conditions used (polyclonal *versus* TCR activation). Importantly, while Notch signaling via OP9‐DL1 resulted in increased chromatin accessibility at the *Gzma* ‐1 kb enhancer (Figure 3g), this did not occur in Rbp‐j deficient CD8^+^ T cells. These results reinforce the notion that Notch signaling drives Gzma expression via initiating chromatin remodeling at the *Gzma* locus into a transcriptionally permissive state, that then enables Gata3 binding.

### Different activation signals result in unique Gata3 binding patterns within activated CD8 ^+^ T cells

Given that Gata3 expression within activated CD8^+^ T cells was determined to be independent of both IL‐4^34^ and Notch signaling (Figures 1 and 3), we next compared genome wide Gata3 binding established after either *in vitro* activation in the presence of IL‐4 or Notch signals, and compared this with patterns established after IAV infection. Gata3 ChIP‐seq was carried out on naïve (CD44^lo^CD62L^hi^) OTIs, after *in vitro* activation in the presence of IL‐4 or Notch signaling (OP9‐DL1), or primary effector OT‐Is isolated directly *ex vivo* 10 days after adoptive transfer and infection with A/HKx31‐OVA (Supplementary figure 1a). Initial comparison of Gata3 peaks identified 148 and 2579 unique Gata3 peaks in naïve or effector OT‐Is induced after IAV infection, respectively, indicating that CD8^+^ T cell activation induces a distinct pattern of Gata3 binding (Supplementary figure 1b). Gata3 specific binding was observed at effector genes such as *Gzma, CCL5* and *Ccr5*, as well as binding at key transcription factors known to underpin CD8^+^ T cell differentiation such as *Batf, Eomes, Runx3* and *Tbx21* (Supplementary table 1; Supplementary figure 2).

To gain an understanding of how distinct extrinsic signals may influence Gata3 binding, a comparison of Gata3 peaks between *ex vivo* effectors and OT‐Is activated *in vitro* in the presence of either IL‐4 or OP9‐DL1 (Notch signals) was carried out. The overlap between all three conditions identified only 324 shared peaks indicative of a core Gata3 program induced by CD8^+^ T cell activation (Supplementary figure 1c). This included increased Gata3 binding to gene loci such as *Gzma* (Supplementary figure 1d), *Il7r*, *CCL5* and *Tbx21* (Supplementary table 1). Interestingly, the greatest overlap of Gata3 binding peaks was found between activated CD8^+^ T cells after OP9‐DL1 signaling and IAV infection (Supplementary figures 1c and 3) suggesting that Notch signaling after IAV infection contributes to Gata3 targeting to key CD8^+^ T cell gene loci. Of interest was the observation that Notch signaling resulted in unique binding of GATA3 to gene loci such as the transcription factors *Myc* and *Myb* (Supplementary table 1).

### Characterization of Gata3 genomic binding sites after distinct modes of CD8 ^+^ T cell activation

**Figure 4:**
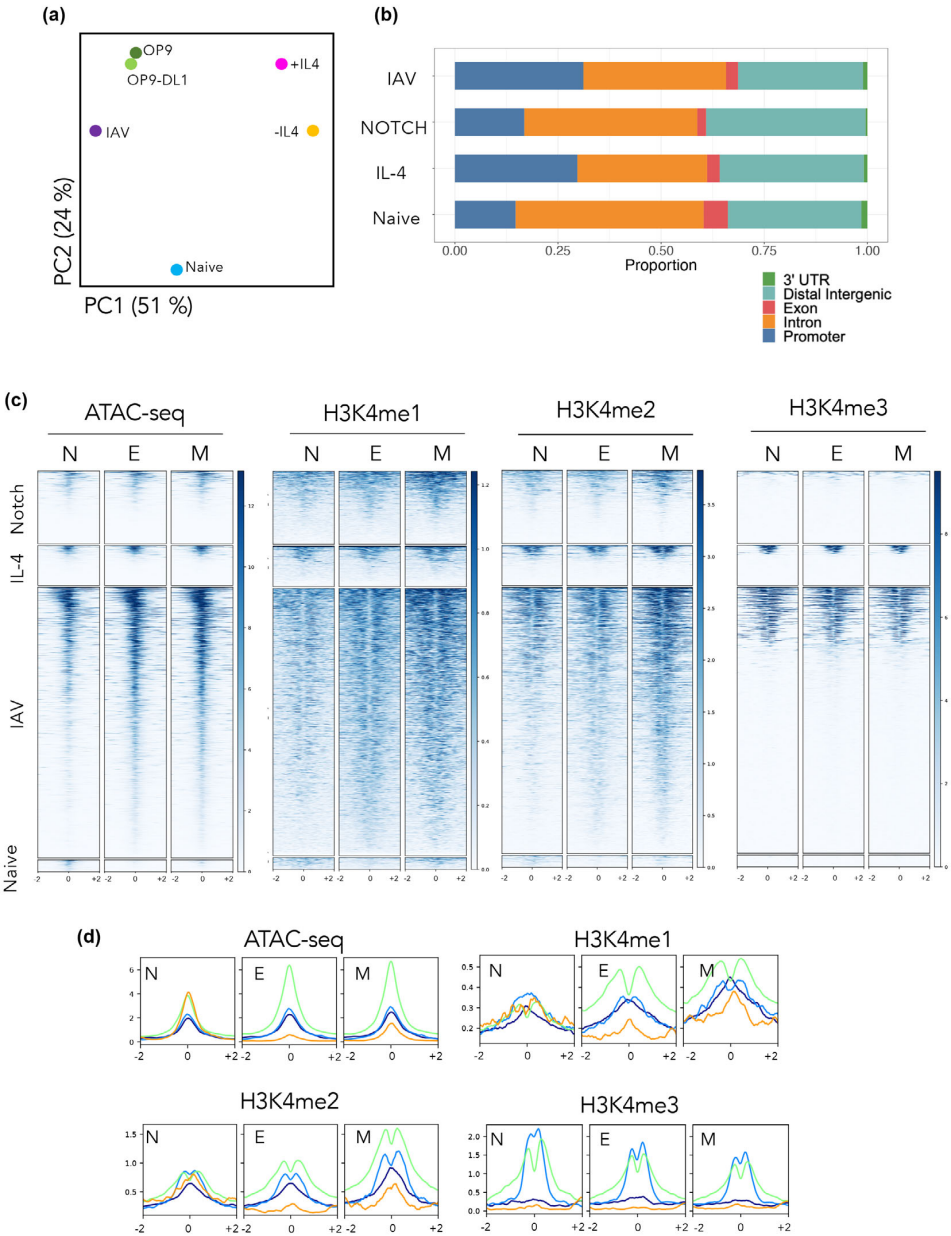
Mapping the chromatin architecture at Gata3 binding sites in differentially activated CD8^+^ T cells. Gata3 ChIP‐seq was carried out on naïve (CD44^lo^CD62L^hi^) OT‐I CD8^+^ T cells, or effector OT‐I CD8^+^ T cells isolated 10 days after IAV infection, or after *in vitro* activation in the presence of either IL‐4 or Notch signaling. **(a)** Principal component analysis was carried out on Gata3 ChIP seq data; **(b)** Gata3 binding was mapped to distinct genomic regions within either naïve OT‐I or activated OT‐I CD8^+^ T cells; **(c)** Regions enriched for Gata3 binding in naïve or activated CD8^+^ OT‐I T cells were overlapped with available ATAC‐seq^49^, or H3K4me1, H3K4me2 and H3K4me4 ChIP‐seq data.^5^, ^11^ Enrichment of chromatin accessibility or histone PTMs around the peak of the Gata3 binding (+/−1 kb) is shown. **(d)** Average peak enrichment around Gata3 peaks for chromatin accessibility (ATAC‐seq), H3K4me1, H3K4me2 and H3K4me3 was assessed for naïve (orange line) OT‐Is, IAV activated OT‐I effector (green line) or effector OT‐I activated *in vitro* in the presence of IL‐4 (light blue) or Notch (dark blue).

To better understand the functional role of Gata3 after CD8^+^ T cell activation, we first assessed the Gata3 ChIP‐seq data by principal component analysis (Figure 4a). Interestingly, the biggest discrepancy in Gata3 binding was observed in PC1, with Il‐4 signals resulting in divergent patterns compared with both Gata3 binding after IAV infection, or activation in the presence of NOTCH (Figure 4a). Gata3 bound genomic regions within each distinct activation state (Figure 4b). A similar Gata3 binding pattern was observed within each activation state with the majority of Gata3 peaks located in either distal, intergenic regions, or within intronic sequences within gene loci (Figure 4b). Gata3 peaks were also enriched, albeit to a lesser extent, at gene promoters, with little or no binding within exons or within 3′UTRs (Figure 4b). Transcriptional enhancers (TEs) can be functionally annotated based on enrichment of histone PTMs such as H3K4me1 (putative enhancers), H3K4me2 (poised or active enhancers), H3K4me3 (a subset of active enhancers) and the extent of chromatin accessibility.^5^, ^46^, ^47^, ^48^ We therefore overlaid Gata3 bound regions identified with previously published ATAC‐seq,^49^ and ChIP‐seq data for H3K4me1, H3K4me2 and H3K4me3 generated from naïve, effector and memory IAV‐specific CD8^+^ T cells.^5^, ^11^ Gata3 binding in each differentiation state largely correlated with regions that had hallmarks of active transcriptional enhancer regulatory elements (increased accessibility, enrichment of H3K4me1 and H3K4me2) (Figure 4c). Interestingly, while both IL‐4 and IAV infection induced Gata3 binding at sites enriched for H3K4me3, typical of gene promoters, Gata3 binding at H3K4me3 sites after Notch signaling was not as evident (Figure 4c). This difference in Gata3 binding patterns and associated histone PTMs was explored in more detail by examining the histone PTM peaks found in naïve (N), effector (E) and memory (M) CD8^+^ T cells located at Gata3 binding sites (Figure 4d). Gata3 binding within naïve CD8^+^ T cells, after Notch or IL‐4 signaling, and after IAV infection, was associated with open chromatin. Gata3 binding after IL‐4 and IAV signaling was located in between histone peaks indicative of Gata3 binding to accessible chromatin between neighboring nucleosomes, associated with transcriptional activation. Interestingly, Gata3 binding after Notch signaling was at regions of H3K4me1, H3K4me2, but not H3K4me3 enrichment. Moreover, there was a central peak of histone PTM enrichment at Gata3 binding sites after Notch signaling. This pattern of histone PTM at TF binding sites is indicative of TFs capable of remodeling chromatin into a more open and transcriptional permissive state.^50^ This aligns with our *in vitro* data suggesting that Notch/Rbp‐j activity remodels chromatin enabling subsequent Gata3 binding (Figure 4).

### Mapping the transition of histone PTM enrichment at Gata3 binding sites after distinct activation signals

**Figure 5:**
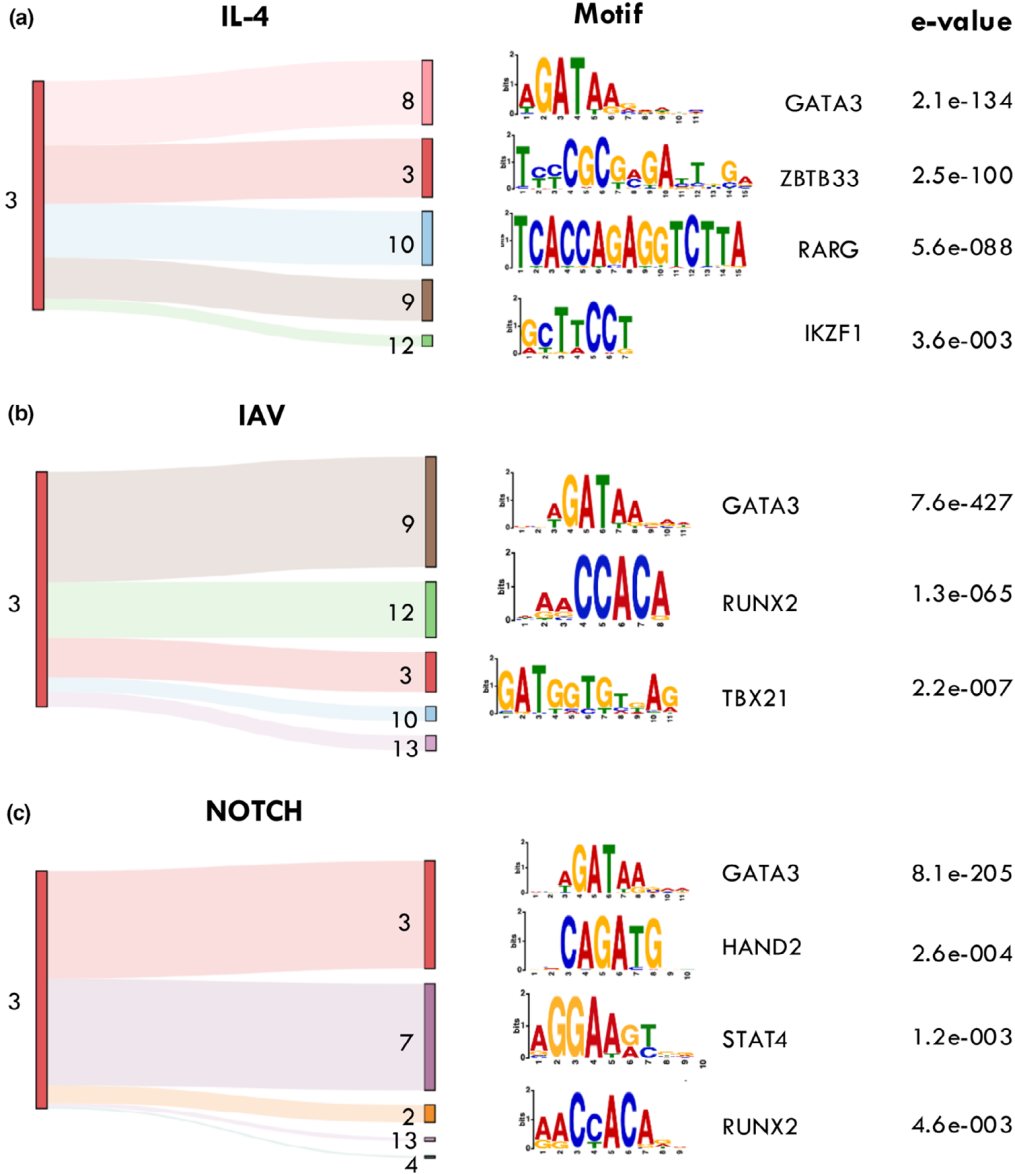
Distinct CD8+ T cell activation signals result in distinct chromatin transitions from the naïve to effector state. GATA binding regions identified in effector CD8^+^ T cells isolated after IAV infection (day 10), or after *in vitro* activation in the presence of IL‐4 or Notch signaling. Distinct chromatin states of these Gata3 bound regions were identified using ChromHMM^51^ by integrating previously generated chromatin accessibility data, H3K4me1, H3K4me2, H3K4me3, H3K27me3 and H3K27Ac ChIP‐seq data. The change in chromatin state from a repressive chromatin structure (emission state 3, Supplementary figure 4) in naïve CD8^+^ T cells to alternative active states was assessed for IL‐4 **(a)**, IAV **(b)** or Notch **(c)** activation. Identification of TFBS associated with Gata3 peaks was done using MEME^65^ with the consensus motif and associated *P*‐value.

We reasoned that the histone PTM dynamics oOld reference number 49 should be new number 51.f transcriptional enhancers bound by Gata3 as CD8^+^ T cells transition from naïve to effector would be distinct after IAV activation *versus* IL‐4 or Notch signaling. Utilizing ChromHMM,^51^ we used both our published histone PTM ChIP‐seq data and a publicly available H3K27Ac data set (GSE111902) to identify and annotate distinct chromatin states at Gata3 binding sites within naïve and effector CD8^+^ T cells (Supplementary figure 4). We initially examined the fate of chromatin states annotated as repressed/poised transcriptional enhancers in naïve CD8^+^ T cells (enriched for H3K27me3, H3K4me1 and, H3K4me2; low chromatin accessibility, low levels of H3K27Ac and H3K4me3, termed emission state 3)^51^ after activation in the presence of IL‐4, Notch or IAV infection (Figure 5). These repressive TEs (emission state 3) transitioned into more active regions after activation with IL‐4 signaling or IAV activation (Figure 5a, b). Interestingly, while IL‐4 induced Gata3 binding at active transcriptional enhancers with a canonical signature (emission state 8), Gata3 binding within IAV induced CD8^+^ T cell effectors was predominantly at active TEs regions that lacked H3K27Ac (emission states 9 and 12). Interestingly, repressed/poised TEs bound by Gata3 after Notch signaling were either unchanged (state 3), or transitioned into an immature chromatin state that was neither repressed nor fully active characterized by a loss of TE histone PTM signatures (H3K34me1/me2), but enriched in H3K27Ac (emission state 7) (Figure 5c). These data suggest that these distinct activation conditions induce quite different chromatin dynamics at Gata3 binding regions in CD8^+^ T cells. To examine whether this dynamic was reflected in other genome differences, we examined the enrichment of binding sites for other transcription factors co‐located with Gata3 binding in these effector states. IL‐4 signaling induced Gata3 binding at regions that also were enriched for the ZBTB33, RARG and IKZF1 TFBS (Figure 5a). In contrast, despite exhibiting differing chromatin dynamics and transition states, Gata3 bound sites after IAV infection or Notch signaling were enriched for accessory TFBS that are associated with CD8^+^ T cell differentiation (STAT4, TBX21 and RUNX2) (Figure 5b, c). Together these data suggest that the receipt of distinct extrinsic signals during T cell activation results in different Gata3 binding patterns and association with distinct TF combinations. Moreover, Notch signaling results in Gata3 binding at sites that become more accessible due to potential pioneering activity of Notch/Rbp‐j. Finally, we examined whether Gata3 peaks observed in naive CD8^+^ T cells, or those activated in the presence of IL‐4 or Notch signaling, or after IAV infection coincided with other published TF binding patterns in T cells (Supplementary figure 5). Unsurprisingly, Gata3 peaks in all four conditions mapped to known regions previously shown to be bound by Gata3 (Supplementary figure 5a–d). However, we did observe distinct binding enrichment for different TFs depending on the differentiation state. For example, Gata3 peaks in naïve CD8^+^ T cells mapped to regions that also known to bind known naïve specific TFs such as TCF‐1 and SATB1 (Supplementary figure 5a). Gata3 peaks in IL‐4 stimulated CD8^+^ T cells exhibited Gata3 targets that were distinct to the other activation conditions. Not surprisingly, this included enrichment of regions that are reported targets of other T_H_2 associated TFs including SATB1^52^ and STAT6^53^ (Supplementary figure 5b). These same regions were also enriched for targets of the histone acetyltransferase, P300, aligning with our earlier analysis demonstrating IL‐4 induced K27Ac enrichment at Gata3 binding sites observed in activated CD8^+^ T cells (Figure 5a, Supplementary figure 4). While Gata3 binding regions induced after Notch signaling, or IAV infection were also somewhat distinct, they both generally had patterns of TF binding for factors known to be involved in CD8^+^ T cell effector differentiation (Supplementary figure 5c, d). This included TBX21, RUNX3, EP300 and STAT5A for IAV effector Gata3 binding regions, and TBX21, RUNX1 and JUN at Notch induced peaks. It was interesting that we demonstrated that RUNX3 is in fact a target of Gata3 after both IAV infection and Notch signaling (Supplementary figure 2), hence it is tempting to speculate that Notch signaling may pioneer the chromatin state of not only effector gene loci, but also the key TFs required for early effector CD8^+^ T cell fate determination.

### Gata3 deficiency results in decreased Gzma expression within virus‐specific CD8 ^+^ T cells

**Figure 6:**
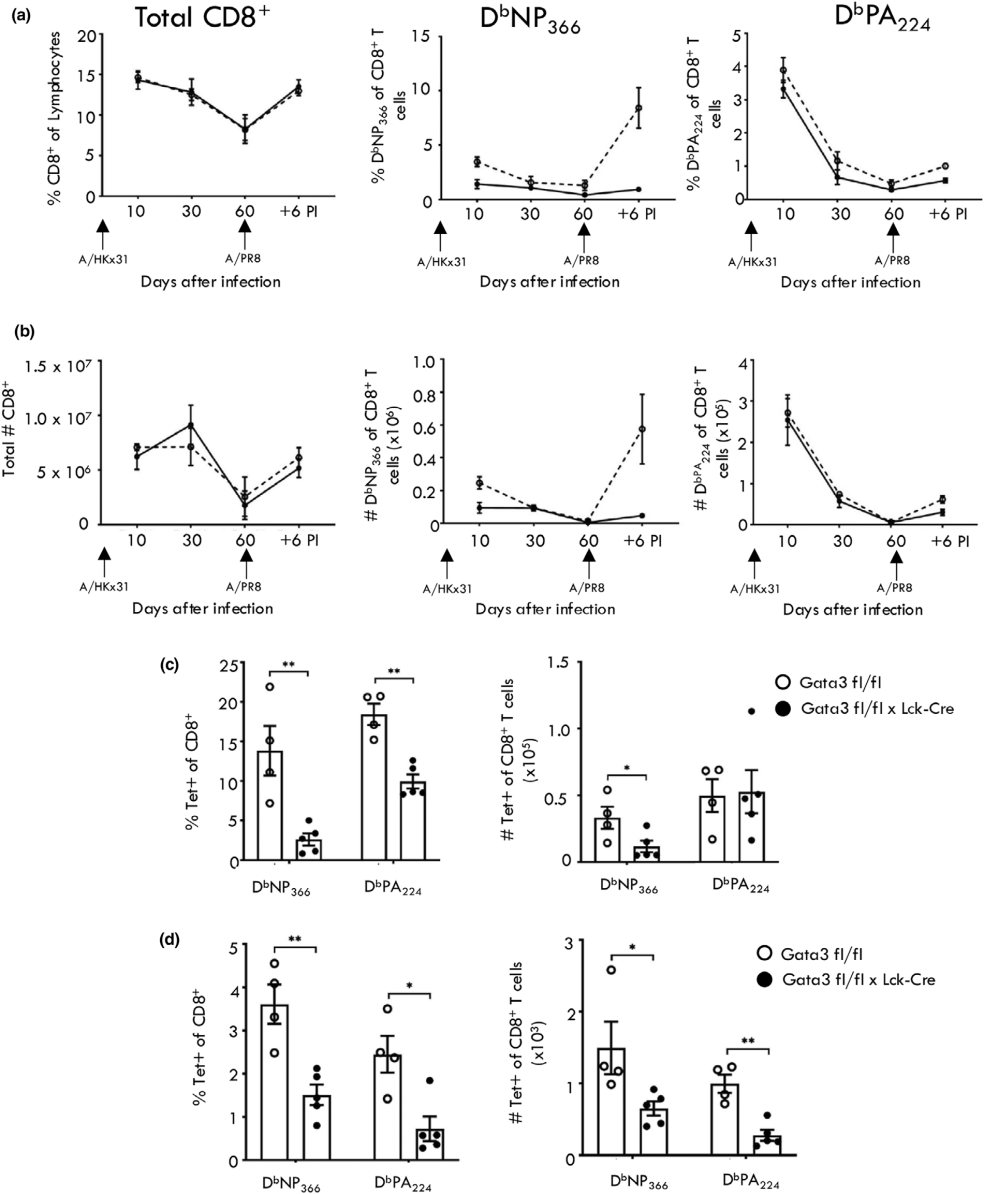
Gata3 deficiency results in reduced IAV‐specific CD8+ T cell primary and immunodominant secondary responses. WT and GATA3KO mice were infected with 10^4^ pfu of A/HKx31 Influenza A virus and the proportion **(a)** and total number **(b)** of splenic CD8^+^ T cells, D^b^NP_366_‐specific and D^b^PA_224_‐specific CD8^+^ T cells assessed at day 10 (primary effector), day 60 (memory) after primary infection, or 7 days after infection with A/PR8‐OVA (secondary). At day 60 after primary infection the proportion and number of tetramer^+^ memory CD8^+^ T cells were enumerated from both the spleen **(c)** and lung parenchyma **(d)**. Data are shown as mean ± s.e.m. (*n* = 3) of four independent experiments. *P < 0.01, **P < 0.001; Kruskal‐Wallis non‐parametric test.

We have previously demonstrated that Gata3 binding occurs at the *Gzma* locus of influenza‐specific CD8^+^ T cells.^34^ Given our data that Notch signaling drives chromatin accessibility, enabling Gata3 binding at the *Gzma* locus, we examined the link between Gata3 and Gzma expression utilizing mice with a T cell specific *Gata3* deletion (GATA3KO). IAV infection of GATA3KO mice generated a lower proportion and fewer absolute numbers of D^b^NP_366_ and D^b^PA_224_ CD8^+^ T cells in both the spleen and bronchoaveolar lavage (BAL) at day 10 after IAV infection, measured by tetramer staining (Figure 6a–c), and demonstrated diminished functionality as measured by intracellular cytokine staining (Supplementary figure 6a). Upon memory formation (day 90 after infection), there was no difference in IAV‐specific CD8^+^ T cell proportions in the spleen (Figure 6a–c); however, there were significantly fewer D^b^NP_366_ and D^b^PA_224_ lung resident T cell memory populations (Figure 6d) in GATA3KO mice compared with WT mice. Despite a similar number and frequency of IAV‐splenic memory CD8^+^ T cells, GATA3KO mice exhibited a significantly diminished secondary effector response compared with WT mice, particularly evident for the immunodominant D^b^NP_366_ specific response (Figure 6a, b).

**Figure 7:**
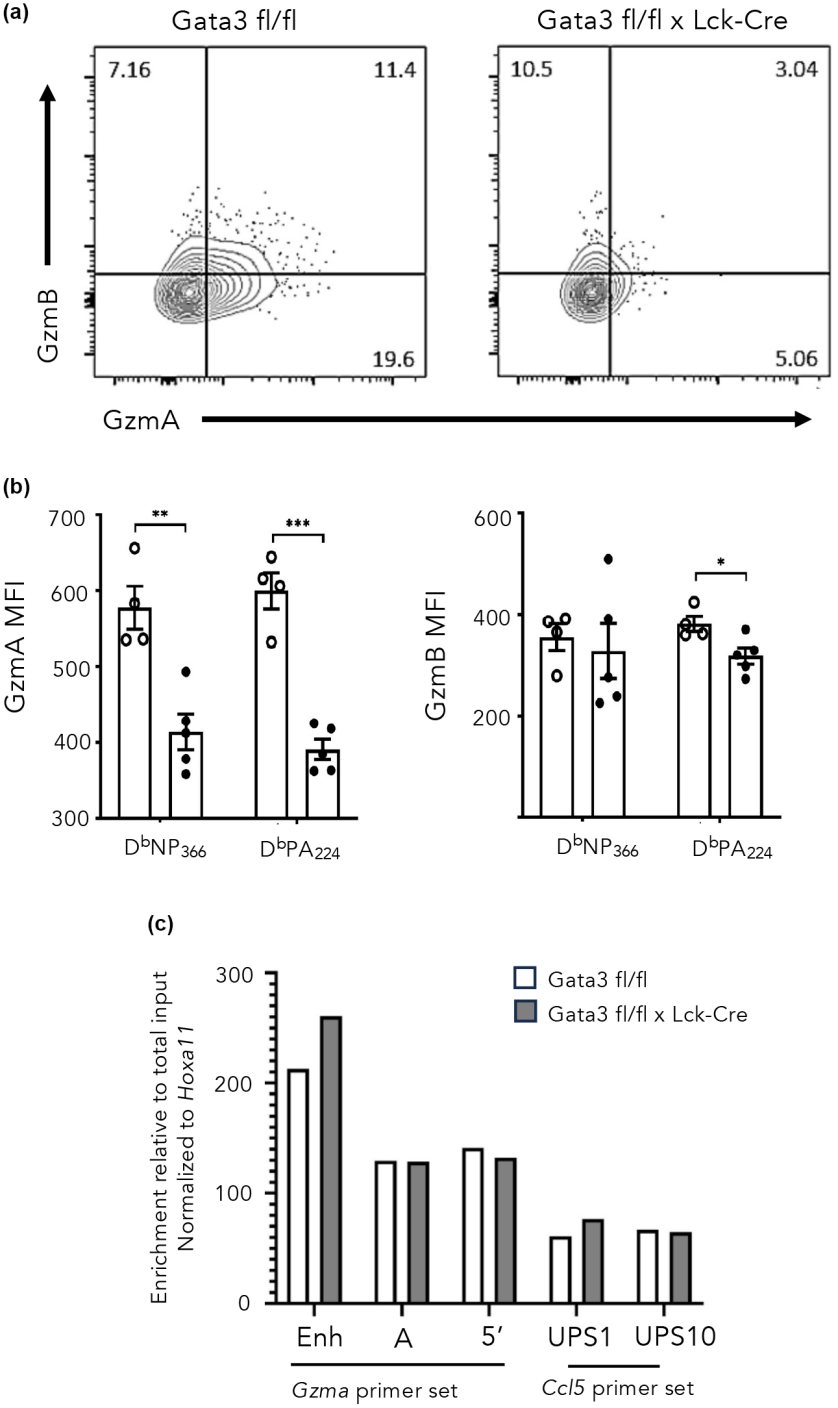
Gata3 deficiency in IAV‐specific CD8^+^ T cells results in diminished Gzma expression despite maintaining chromatin accessibility. WT and GATA3KO mice were infected with 10^4^ pfu of A/HKx31 Influenza A virus and BAL fluid collected at day 10 post‐infection. **(a)** Proportion and **(b)** MFI of Gzma and Gzmb was assessed by intracellular staining for **(a, b)** CD8^+^D^b^NP_366_‐specific and **(b, c)** D^b^PA_224_‐specific CD8^+^ T cells. **(c)** CD8^+^D^b^NP_366_‐specific and D^b^PA_224_‐specific CD8^+^ T cells from the BAL of day 10 IAV infected mice, were sorted, pooled and subjected to FAIRE. Chromatin accessibility was assessed using primers specific for *Gzma enhancer, Gzma TSS (set A and B)* and *CCL5* TSS (Ups1 and Ups10). Chromatin accessibility was determined relative to the *Hoxa11* locus. *P < 0.01, **P < 0.001; ***P < 0.002; Kruskal‐Wallis non‐parametric test.

Given that we have previously shown that IAV‐specific effector CD8^+^ T cells isolated from the BAL express the highest levels of Gzma,^2^, ^34^ we compared Gzma expression by D^b^NP_366_ and D^b^PA_224_ specific CD8^+^ T cells from the BAL isolated 10 days after infection (Figure 7). Gata3 deficient IAV‐specific CD8^+^ T cells expressed a lower proportion of Gzma (Figure 7a), and lower overall levels of Gzma (Figure 7b) compared with WT IAV‐specific CD8^+^ T cells. While there was a slightly small proportion of Gzmb^+^ IAV‐specific CD8^+^ T cells from GATA3 KO mice, there was no difference in the levels of Gzmb expression (Figure 7a, b). Importantly, there was no difference in chromatin accessibility at the *Gzma* or locus between WT and Gata3 KO IAV‐specific CD8^+^ T cells isolated from BAL (Figure 7c). This supports the concept that extrinsic factors, such as Notch, play a role in initially remodeling the chromatin structure at the *Gzma* locus, thus enabling Gata3 binding and transcriptional activation.

## DISCUSSION

We had previously demonstrated that Gzma was a target of Gata3 within activated CD8^+^ T cells after IAV infection.^34^ Importantly, this was independent of IL‐4 mediated signaling suggesting an alternative pathway for Gata3 regulation of Gzma expression. Notch signals have previously been shown to act as an alternative signaling pathway resulting in Gata3 upregulation and T_H_2 CD4^+^ T cell lineage commitment.^42^, ^43^ We therefore reasoned that Notch signaling on CD8^+^ T cell drives Gata3 regulation of Gzma expression. This hypothesis was supported by our ChIP‐seq data demonstrating that Gata3 was bound to the *Gzma* locus in not only IAV‐specific CD8^+^ T cells, but in activated CD8^+^ T cells that had received both IL‐4 and Notch signals. Importantly, our data demonstrated that Gata3 expression alone was not sufficient for Gzma expression. Importantly, while TCR signaling was sufficient for Gata3 upregulation, Gzma expression was only induced when Notch signals were present. The role of Notch signaling appears to act on chromatin in a Rbp‐j dependent manner, resulting in increased chromatin accessibility at TEs within the *Gzma* locus, that then enables Gata3 binding to drive Gzma expression. Hence, our data suggest that Notch signaling renders TEs linked to CD8^+^ T cell effector genes poised for TF binding and enabling subsequent transcriptional activation (Figure 7). These data highlight how extrinsic signals generated during an acute immune response play a role in helping to promote CD8^+^ T cell responses by restructuring the chromatin landscape that can then be read by key TFs to drive effector T cell differentiation.

Two earlier studies utilized Gata3 deficient T cell models to demonstrate that Gata3 upregulation after T cell activation is key for the acquisition and maintenance of T cell effector function and differentiation in response to tumor or LCMV infection.^32^, ^33^ We demonstrated that Gata3 deficiency also resulted in diminished IAV‐specific CD8^+^ T cell primary and secondary effector responses, supporting these earlier studies. The study by Wang and colleagues reported a defect in IL‐7 receptor expression with Gata3 deficiency leading to a loss of memory T cell homeostasis. While our ChIP‐seq data identified the *Il7ra* locus as a Gata3 target after IAV infection (Supplementary table 1), the inability of Gata3 deficient CD8^+^ T cells to mount a significant recall response was not due to differences in memory precursors. However, in the same study, Wang and colleagues also identified that c‐MYC, a key regulator of cell cycle initiation was a target of Gata3 binding after T cell activation.^33^ They also showed Gata3 deficiency and the lack of c‐MYC activation. Notch signaling resulted in Gata3 binding to cMyc (Supplementary table 1) within the *in vitro* activated CD8^+^ T cells. When combined with our observation that IAV‐specific primary and secondary effector responses were reduced with Gata3 deficiency, it is tempting to speculate that early Notch signaling results might result in chromatin remodeling of the Myc locus, enabling Gata3 dependent binding and c‐Myc activation to promote sustained CD8^+^ T cell expansion.

Given our data linking Notch dependent chromatin priming for Gata3 binding at the *Gzma* locus, a key question is just how broad is this mode of activation? We observed that Gata3 was bound to the *Il7ra* locus in both naïve and effector CD8^+^ T cells. This suggests that there is no need for Notch dependent priming of the *Il7ra* locus upon T cell activation. Interestingly, we did observe Gata3 binding to the *Myc* locus after both IAV infection and activation in the presence of Notch signaling, but not IL‐4 signaling. It is tempting to speculate that the ability to engage c‐Myc after activation does in fact require Notch‐dependent chromatin remodeling and subsequent Gata3 binding. It remains to be determined whether such remodeling is needed for rapid transcriptional activation after TCR activation or is also required to maintain the transcriptional responsiveness of c‐MYC within memory CD8^+^ T cells.

Activation of dendritic cells with TLR agonists upregulates Delta‐like ligand expression, with TCR activation resulting in upregulation of Notch1 and 2 receptors on activated CD8^+^ T cells. Hence, the receipt of Notch signals is likely occurring at the same time as T cell activation after infection. Moreover, Notch deficient CD8^+^ T cells fail to fully engage an effector CD8^+^ transcriptional program and show decreased expression of Gzma compared with wildtype CD8^+^ T cells.^38^ While this study also showed that Rbp‐j deficiency also resulted in diminished effector CD8^+^ T cell responses linking Notch signaling with Rbp‐j activity, the precise mechanism of action and the link to TFs, such as Gata3, were not examined fully. Hence, our data suggest a potential link where extrinsic signaling via Notch results in a poising of the CD8^+^ T cell genome enabling binding of Gata3 to drive effector CD8^+^ T cell effector gene expression. A separate study also demonstrated that Notch signaling is required for the acquisition of CD8^+^ T cell function.^39^ Moreover, this study showed that over expression of Ncid in T cell hybridomas directly interacted with Cre binding protein 1 (Creb1) resulting in binding to and transcriptional activation of the *Gzmb* locus. Hence, it appears that Notch signaling can have both direct and indirect impacts on the transcriptional activation of CD8^+^ T cell effector gene expression. We are currently examining this question with Rbp‐j deficient mouse models to delineate the molecular mechanisms that underpin Notch regulation of CD8^+^ T cell responsiveness.

There was minimal overlap of Gata3 binding patterns within CD8^+^ T cells activated after IAV infection, or after *in vitro* activation in the presence of either IL‐4 or Notch signals. While common targets of Gata3 binding in all three conditions did include gene loci such as *Gzma, Tbx21, CCL5* and *Runx3*, there was little else related to engagement of CD8^+^ T cell effector programs to indicate the engagement of a core CD8^+^ T cell program after Gata3 binding. This likely reflects the fact that extrinsic signals received by an activated CD8^+^ T cell likely results in quite distinct chromatin landscapes that are then interpreted in different ways by common TFs, such as Gata3. It was therefore of interest that there was a greater degree of overlap of Gata3 binding between IAV infected CD8^+^ T cells and those activated in the presence of Notch signals. This suggests that perhaps Notch is acting, presumably early after activation, to help establish Gata3 binding patterns associated with conventional effector CD8^+^ T cell differentiation. This is further supported by the observation that IAV and Notch activated Gata3 binding sites were associated with enrichment of the same secondary TFBS, namely Runx2. Runx2 has previously been reported to be important for memory precursor generation and persistence of memory CD8^+^ T cells.^50^ Although a separate study did not find a significant role for Runx2 in CD8^+^ T cell response to infection.^54^ Nevertheless, it is tempting to speculate that Gata3 and Runx2 may combine to help shape CD8^+^ T cell responses to infection.

Together, our data demonstrate that while TCR signals are sufficient for expression of key transcription factors, the receipt of extrinsic signals during activation that then sculpt the chromatin landscape to enable TF binding is required for acquisition of effector function. This two‐step process could perhaps be considered an epigenetic checkpoint. Much like the role of CD28 costimulation which is a key go signal for T cell activation and is provided by activated DCs after infection, the ability of TFs to drive optimal effector CD8^+^ T cell differentiation might in some cases only be enabled if signals received from either accessory cells such as activated dendritic cells, or other inflammatory signals prepare the chromatin accordingly to allow TF binding. In this way, the acquisition of potent effector function, such as Gzma expression, is tightly controlled and is only engaged in the right circumstances (i.e. in response to infection). Understanding the interplay between extrinsic signals, such as Notch, among others, and chromatin remodeling leading to effector function could provide insights into improved therapeutic strategies against different cancers or infections, help to understand how signals in the tumor microenvironment might lead to immune escape or perhaps provide insights into immunopathology caused by inappropriate CD8^+^ T cell responses.

## METHODS

### Mice, viruses and infection

Ly5.2^+^ C57BL/6, congenic Ly5.1^+^ OT‐I (OT‐I), Gata3^tm1.1Mbu^/J (Gata3 fl/fl) and Rbpj^tm1Hon^ (Rbpj fl/fl, Riken BRC, Japan) mice were bred and housed under specific‐pathogen free conditions at the Animal Research Laboratory, Monash University. Gata3 fl/fl and Rbpj fl/fl mice were crossed onto the Lck‐Cre transgenic mouse line (B6.Cg‐Tg(Lck‐cre)548Jxm/J, The Jackson Laboratories, Bar Harbor, ME, USA) mice to generate mice with a T cell conditional knockout of *Gata3* ^55^ and *Rbpj*,^56^ respectively (Gata3^fl/fl^ Lck‐Cre and Rbp‐j Lck‐Cre). For primary infections, naive mice were anesthetized and infected intranasally (i.n.) with 1 × 10^4^ plaque‐forming units (PFU) of A/HKx31 (H3N2) influenza A virus (IAV), or with 1 × 10^4^ PFU of recombinant HKx31 IAV engineered to express the OVA_257‐264_ peptide (HKx31‐OVA) in the neuraminidase stalk (Jenkins 2006). For secondary infections (> day 60 post‐HKx31 infection), mice were anesthetized and infected i.n. with 1 × 10^3^ PFU of PR8 (H1N1) IAV. All experiments were conducted according to approval obtained from the Monash University Animal Ethics Committee.

### Adoptive transfer, tissue sampling and cell sorting

For adoptive transfers, 1 × 10^4^ naive OT‐I cells from pooled lymph nodes (LNs) were injected intravenously into C57BL/6 recipient mice 24 h prior to infection with HKx31‐OVA. The Ly5.1^+^ OT‐I cells were isolated from spleens at 7 days post‐infection. For cell sorting, pooled spleen and LN preparations from OT‐I, C57BL/6 or Rbp‐j Lck‐Cre mice were stained with anti‐CD8⍺‐Pacific Blue (53–6.7; BD BioSciences, Franklin Lakes, NJ, USA), anti‐CD44‐PE‐Cy7 (IM7; eBioscience) and anti‐CD62L‐BV605 (MEL‐14; BD BioSciences) to isolate naive (CD44^lo^CD62L^hi^) CD8^+^ T cells, which were sort purified using a FACSAria or Influx cell sorter (BD Biosciences). Following infection of Gata3 fl/fl and Gata3 fl/fl Lck‐Cre mice, spleens were harvested at days 10, 30, > 60 and day 6 post‐secondary infection, bronchiolar lavage fluid (BAL) was harvested at day 10 and lungs were harvested at day 60. Lungs were perfused prior to harvesting.

### Cell lines and cell culture

Control OP9 stromal cells and OP9 cells transfected with the Notch DL1 ligand (OP9‐DL1) [gifted from Professor Dale Godfrey (Department of Microbiology and Immunology, The University of Melbourne)] were cultured in complete DMEM media (10% FCS, 2 mm L‐glutamine, 1 mm sodium pyruvate, 100 μm non‐essential amino acids, 5 mm HEPES, 55 μm β‐mercaptoethanol, 100 U L^−1^ penicillin and 100 μg mL^−1^ streptomycin) and maintained at 1 × 10^5^ cells mL^−1^. The mutu‐DC cells (obtained from Professor Sammy Bedoui, Department of Microbiology and Immunology, The University of Melbourne) were cultured in IMDM media supplemented with 8% FCS, 55 μm β‐mercaptoethanol and penicillin and streptomycin. Prior to OT‐I CD8^+^ T cell stimulation, 2 × 10^6^ cells mL^−1^ of mutu‐DCs were stimulated overnight with 1 μm CpG. The cells were then incubated with 1 μm OVA_257‐264_ peptide for 1 h to obtain peptide‐loaded mutu‐DCs. For OT‐I and OP9 co‐cultures, 1 × 10^5^ freshly sorted naive CD8^+^ OT‐I cells were stimulated with 1 × 10^4^ peptide‐loaded mutu‐DCs (10:1 ratio) and cultured on a monolayer of 1 × 10^5^ OP9 or OP9‐DL1 cells in complete RPMI media (cRPMI, RPMI; Gibco, Waltham, MA, USA) with 10% FCS (Gibco), 2 mm L‐glutamine (Gibco), 1 mm sodium pyruvate (Gibco), 100 μm non‐ essential amino acids (Gibco), 5 mm HEPES (Gibco), 55 μm β‐mercaptoethanol (Gibco), 100 U L^−1^ penicillin and 100 μg mL^−1^ streptomycin (Gibco) with 10 U mL^−1^ rhIL‐2 (Roche Diagnostics, Basel, Switzerland). For cultures investigating blocking of γ‐secretase activity, the cells were also treated with 5 μm of the γ‐secretase inhibitor DAPT or DMSO vehicle control. At days 3 and 4, the cells were re‐plated onto fresh monolayers of OP9 or OP9‐DL1 cells with replenished media, IL‐2 and treatments. The OT‐I cells were analyzed at days 4 and 6. For C57BL/6‐ and Rbp‐j fl/fl x Lck‐Cre‐derived CD8^+^ T cell and OP9 co‐cultures, 1 × 10^5^ freshly sorted naive CD8^+^ T cells were stimulated with 1 × 10^5^ Dynabeads™ Mouse T‐Activator CD3/CD28 microbeads (Thermo Fisher, Waltham, MA, USA) and cultured on a monolayer of 1 × 10^5^ OP9 or OP9‐DL1 cells in cRPMI with 10 U mL^−1^ rhIL‐2. Microbeads were removed at day 3 and the cells were re‐plated onto fresh monolayers of OP9 or OP9‐DL1 cells with replenished media and IL‐2. The CD8^+^ T cells were analyzed at day 4. For CD8^+^ OT‐I cells stimulated in the presence of IL‐4, freshly sorted naive CD8^+^ OT‐I cells were stimulated with CpG‐activated, OVA_257‐264_ peptide‐loaded Mutu‐DCs at a 10:1 OT‐I to DC ratio and supplemented with 25 ng mL^−1^ mouse recombinant IL‐4, 1 μg mL^−1^ anti‐IFN‐γ and 10 U mL^−1^ rhIL‐2 in cRPMI. The cells were re‐plated with fresh media and supplements at day 3 and analyzed at day 6.

### Tetramer, protein surface and intracellular staining and flow cytometry

H2D^b^NP_366_ and H2D^b^PA_224_ monomers were tetramerized using streptavidin‐conjugated phycoerythrin (PE) (Invitrogen, Waltham, MA, USA) as described previously.^2^ Prior to surface antibody staining, the cells were stained with D^b^NP_366_‐PE or D^b^PA_224_‐PE tetramer for 1 h at room temperature. The cells were then stained with surface antibodies anti‐CD4‐AF700 (GK1.5; BioLegend, San Diego, CA, USA) and anti‐CD8⍺‐BUV395 (53–6.7; BD BioSciences). For intracellular cytokine staining, the cells were stimulated for 5 h with 1 μm NP_366‐375_ or PA_224‐233_ peptides in the presence of 10 U mL^−1^ rhIL‐2 and 1 μg mL^−1^ GolgiPlug (BD Biosciences). Following surface antibody staining, the cells were fixed and permeabilized using the BD Cytofix/Cytoperm fixation/permeabilization kit (BD Biosciences) according to the manufacturer's instructions. The cells were then stained with anti‐Ifn‐γ‐FITC (XMG1.2; BD Biosciences) and anti‐Tnf‐APC (MP6‐XT22; BioLegend). For intracellular transcription factor and Granzyme staining, the cells were fixed and permeabilized using the eBioscience Foxp3/Transcription Factor Staining Buffer Set (Invitrogen) according to the manufacturer's instructions. The cells were then stained with anti‐Gata3‐eFluor 660 (TWAJ; eBioscience, San Diego, USA), anti‐Gzma‐FITC (3G8.5; Santa Cruz Biotechnology, Dallas, USA) and anti‐Gzmb‐Pacific Blue (GB11; BioLegend). Live cells were discriminated using LIVE/DEAD Fixable Aqua Dead Cell Stain (Life Technologies, Waltham, MA, USA). The data were acquired using a BD LSR Fortessa X‐20 flow cytometer (BD Biosciences) and FACSDIVA software, and analysis was performed using FlowJo V10.

### Chromatin immunoprecipitation (ChIP)

A total of 2 × 10^5^ to 4 × 10^6^ cells were crosslinked with 0.6% (for histone modification ChIP) or 6% (for Gata3 ChIP) formaldehyde, sonicated and immunoprecipitated with ChIP grade antibodies (5 μg anti‐H3K4me3, 5 μg anti‐H3K9Ac and 10 μg anti‐Gata3) and Protein A magnetic beads (Millipore Sigma, Burlington, MA, USA). Total input and no‐antibody control for each sample was included. Immuno‐precipitated DNA was purified, and quantitative PCR was performed on the CFX‐Connect Real‐Time System (Biorad, Hercules, CA, USA) with Power SYBR Green PCR Master Mix (Applied Biosystem, Waltham, MA, USA) and primers spanning the region of interest. The PCR cycle threshold (Ct) values were converted to copy number (#copies = 10^5^/2^Ct‐17^), background immunoprecipitation was subtracted (no‐antibody control) and the values were normalized to total input. For ChIP‐sequencing, DNA was prepared as for ChIP, and then sequenced on an Illumina HiSeq2000 (Illumina, San Deigo, CA, USA).

### Formaldehyde‐assisted isolation of regulatory elements (FAIRE)

A total of 3 × 10^5^ to 1 × 10^6^ cells were crosslinked with 0.6% formaldehyde, sonicated and open chromatin sites were extracted using phenol: chloroform: isoamyl (25:24:1) (Sigma‐Aldrich, St Louis, MO, USA). DNA was quantified by PCR and data were analyzed and normalized to total input as described for ChIP. For FAIRE analysis of CTLs *ex vivo*, BAL fluid was taken from Gata3^fl/fl^ and Gata3^fl/fl^ × Lck‐Cre mice at day 10 post‐HKx31 infection and pooled CD8^+^ D^b^NP_366_^+^ and D^b^PA_224_^+^ cells were isolated by FACS before being subjected to analysis.

### Chromatin state, motif enrichment and transcription factor binding overlap analyses

The Gata3 peak calls unique to each condition were obtained using bedtools^57^ (v2.31.1). Signal enrichment around the peak regions was computed using deepTools^58^ (v3.5.2^59^) computeMatrix and visualized through heatmaps and profile plots generated with deepTools plotHeatmap. Genomic regions type annotations for the peaks were added using the ChIPseeker (v1.32.0) package.^59^ For chromatin state analysis, BAM files from histone ChIP‐seq and ATAC‐seq datasets were binarised using ChromHMM^51^ (v1.24) BinarizeBAM. Subsequent model learning, segmentation, and the creation of genome browser‐compatible bed files were performed with ChromHMM LearnModel. Emission probabilities and fold enrichments in condition‐specific Gata3 peaks were visualized using ComplexHeatmap^60^ (v2.12.1). State‐specific transitions for individual cell type pairs were precomputed using bedtools and visualized in an interactive app developed with Shiny (v1.7.2, Chang, W., *Shiny: Web Application Framework for R*. https://CRAN.R‐project.org/package=shiny) and Plotly (v4.10.0). To determine motif enrichment in condition‐specific Gata3 peaks, we extracted genome sequences 100 bp upstream and downstream of the peak center using bedtools and awk. MEME‐Chip^61^ (v5.5.3) was then used in command‐line mode to identify motif enrichment in the JASPAR 2020 core vertebrate database.^62^ The condition‐specific peaks were submitted to the CistromeDB^63^ toolkit for transcription factor binding overlap analysis, and plots depicting GIGGLE^64^ scores were generated.

### Statistical analysis

Statistical analyses were conducted using the Kruskal‐Wallis non‐parametric test on Graphpad Prism software. Error bars indicate s.e.m. and *n* values represent biological replicates.

## AUTHOR CONTRIBUTIONS

**Jessie O'Hara:** Conceptualization; formal analysis; investigation; methodology; project administration; supervision; visualization; writing – original draft. **Pushkar Dakle:** Conceptualization; formal analysis; investigation; methodology; resources; software; visualization; writing – review and editing. **Michelle Ly Thai Nguyen:** Conceptualization; formal analysis; investigation; methodology; visualization; writing – review and editing. **Adele Barugahare:** Formal analysis; methodology; software; visualization. **Taylah J Bennett:** Formal analysis; investigation; writing – review and editing. **Vibha AV Udupa:** Formal analysis; investigation; methodology; validation; visualization. **Nicholas Murray:** Conceptualization; formal analysis; investigation; methodology; validation; visualization. **Gemma Schlegel:** Formal analysis; investigation; methodology. **Constantine Kapouleas:** Formal analysis; investigation; methodology. **Jasmine Li:** Formal analysis; investigation; methodology; validation; visualization. **Stephen J Turner:** Conceptualization; formal analysis; funding acquisition; investigation; methodology; project administration; resources; supervision; visualization; writing – original draft; writing – review and editing. **Brendan E Russ:** Conceptualization; formal analysis; investigation; methodology; project administration; supervision; visualization; writing – review and editing.

## CONFLICT OF INTEREST

The authors declare no conflict of interest.

## Supporting information


**Supplementary figures** **1**–**6**

Supplementary table 1

## Data Availability

Data are available upon request from the corresponding authors.
