# Supplementary material for: Notch dependent chromatin remodeling enables Gata3 binding and drives lineage specific CD8 + T cell function
Source: Immunol Cell Biol. 2025 Feb 26;103(4):365–82. doi: 10.1111/imcb.70002 (PMC11964803; doi:10.1111/imcb.70002)
Supplement: Supplementary file 1 — Supplementary figures 1–6 [file IMCB-103-365-s001.pdf]

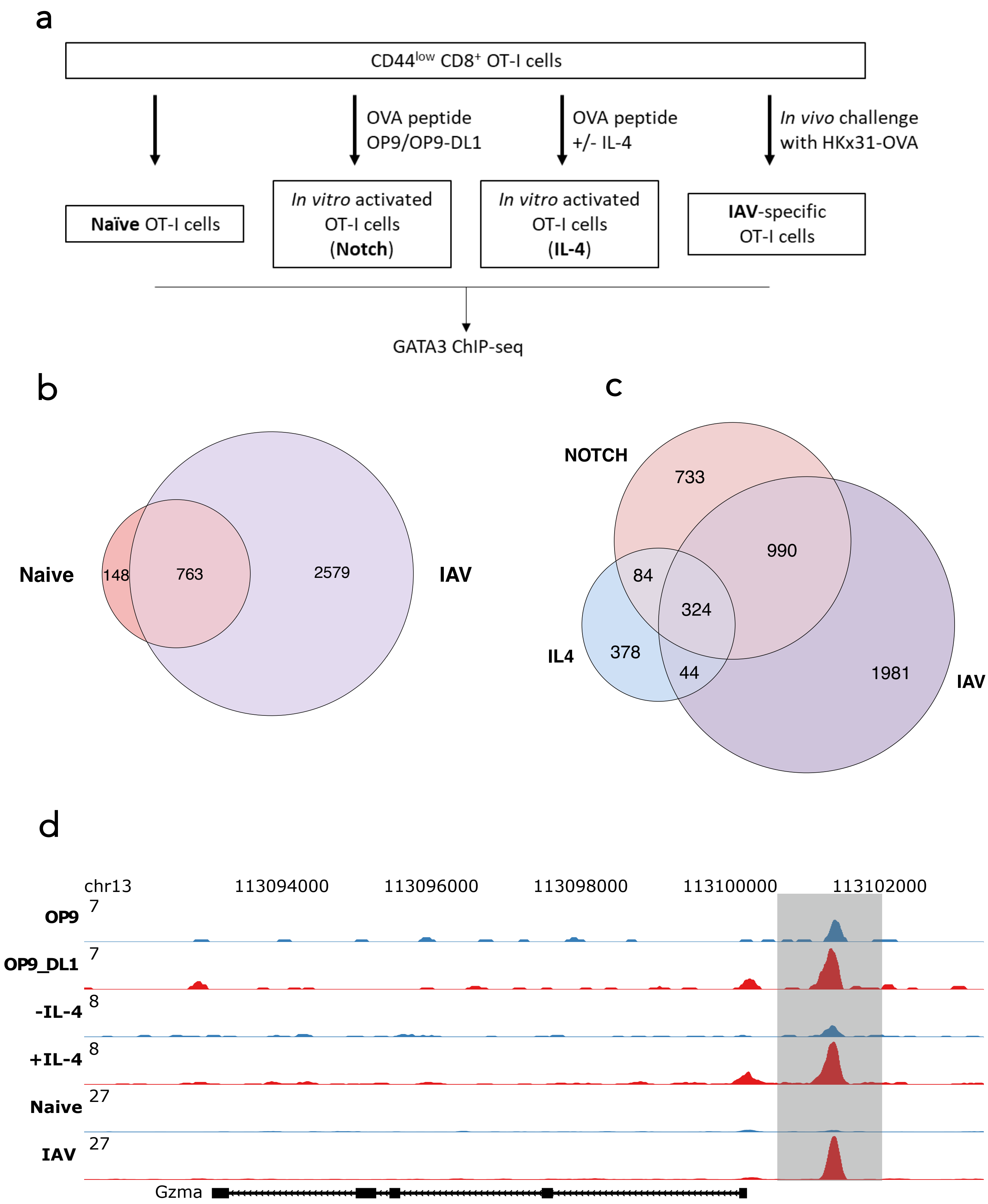

Supplementary figure 1. (**a**) Schematic showing experimental design for Gata3 ChIP-seq on activated CD8⁺ T cells. (**b**) Comparison of Gata3 peaks called between naïve and tetramer⁺ (D^bNP_{366} and D^bPA_{224}-specific) CD8⁺ T cells isolated at day 10 after primary, intranasal A/HKx31-OVA infection, or (**c**) between *in vitro* activated CD8⁺ T cells in the presence of IL-4, Notch signals, compared to IAV-specific CD8⁺ cells. (**d**) Genome browser tracks for Gata3 binding at the *Gzma* locus for different activated CD8⁺ T cell populations.

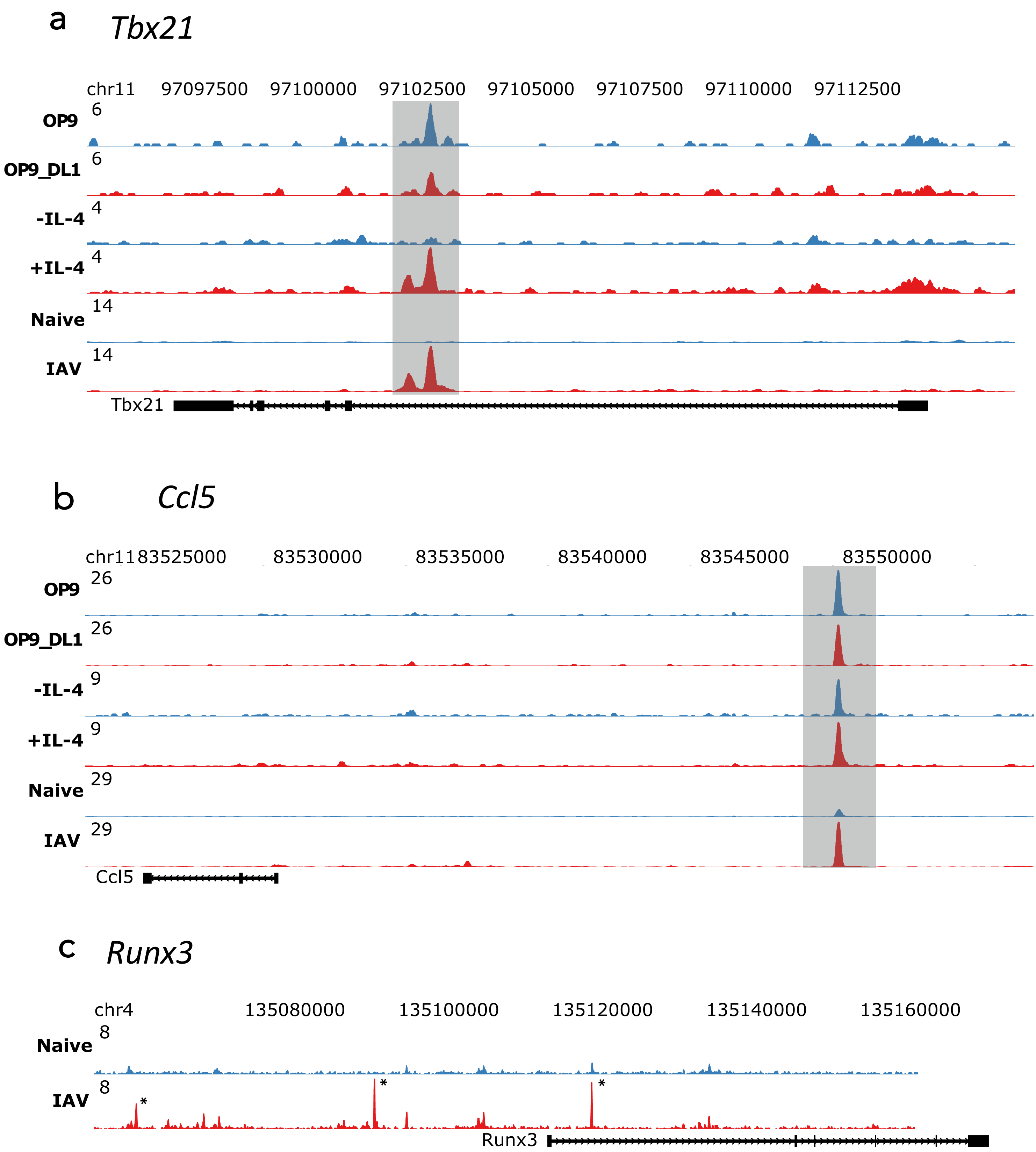

Supplementary figure 2. Genome browser tracks for Gata3 binding at the *Tbx21* (**a**), *Ccl5* (**b**) and *Runx3* (**c**) locus for different activated CD8$^+$ T cell populations.

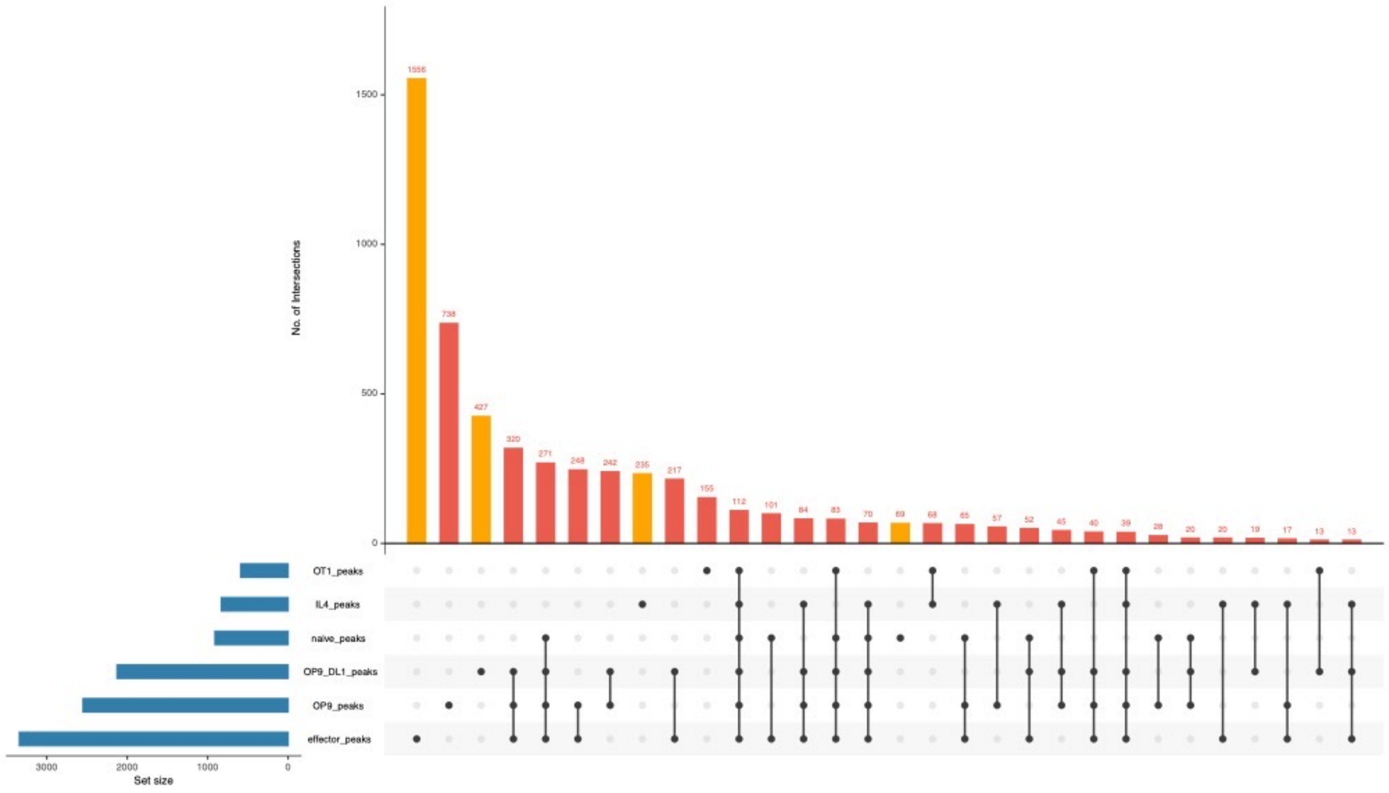

Supplementary figure 3. Overlap in Gata3 binding patterns between the distinct *in vitro* and *ex vivo* activated CD8⁺ T cell subsets. Shown is the number of peaks for each grouping.

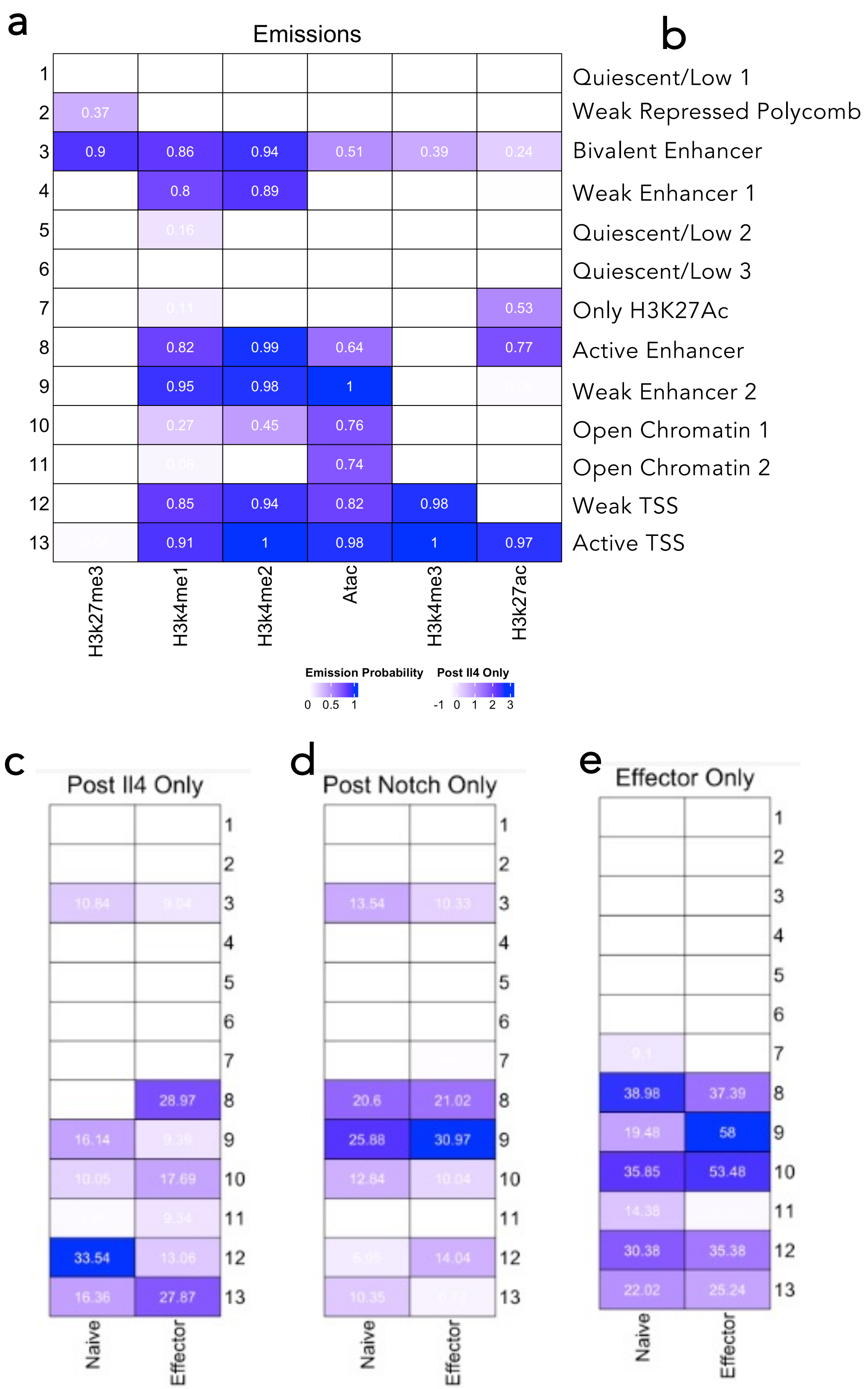

Supplementary figure 4. Visualising chromatin dynamics at Gata3 binding sites activated CD8⁺ T cells (**a**) Gata3 peaks were identified from ChIP-seq data described in Figure 4 and Supplementary figure 1. ChromHMM was utilized to integrate ChIP-seq data for H3K27me3, H3K4me1, H3K4me2, H3K4me3, ATAC-seq and H3K27Ac and generate distinct chromatin emission states based on the combination of histone PTMs at specific genome regions. (**b**) These emission states are then designated to specific genome regulatory functional elements. (**c** – **e**) the change in emission state enrichment and their functional role was compared between naïve CD8⁺T cells, and CD8⁺ T cells activated in the presence of IL-4 (**c**), Notch (**d**) or effector CD8⁺ T cells isolated directly *ex vivo* at the peak of primary IAV infection (**e**).

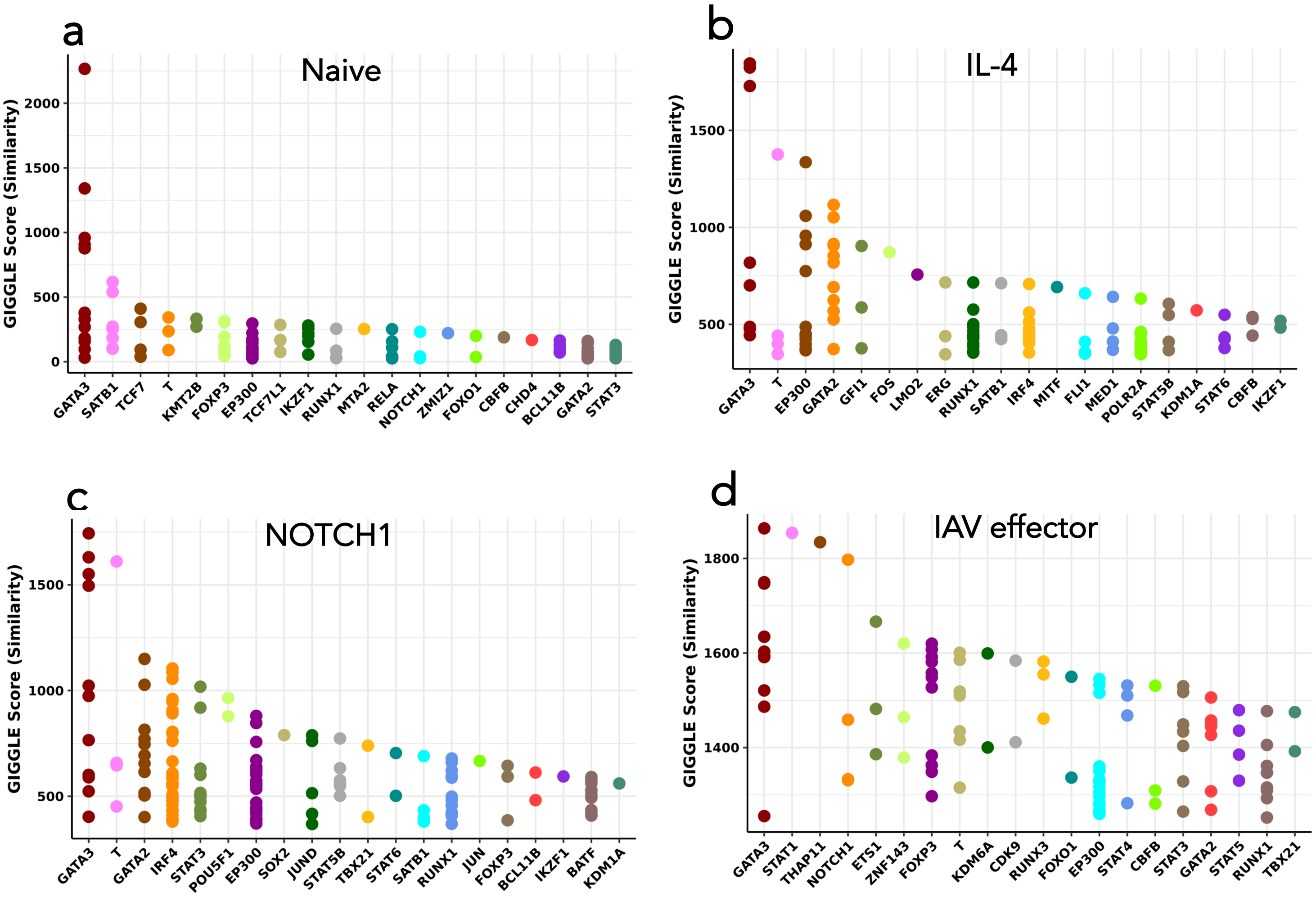

Supplementary figure 5. Overlap in TFBS for with Gata3 binding. TFBS enrichment of motifs associated with Gata3 binding in naïve $CD8^+$ T cells (**a**), or $CD8^+$ T cells activated in the presence of Notch (**b**), IL-4 (**c**), or effector $CD8^+$ T cells isolated directly *ex vivo* at the peak of primary IAV infection (**d**). GIGGLE was utilized to identify overlap with Gata3 sites with other TFBS from publicly available ChIP-seq data.

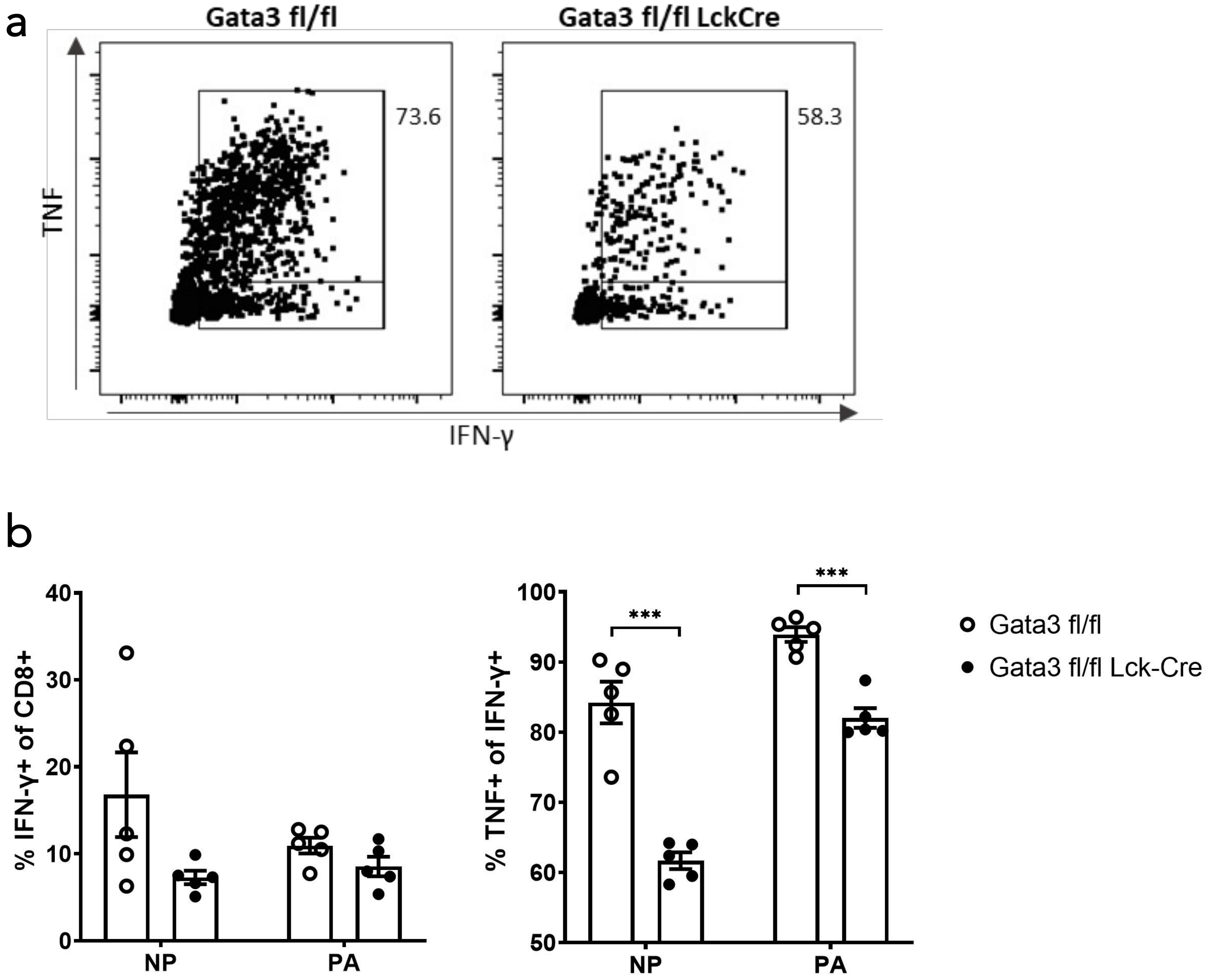

Supplementary figure 6. Gata3$^{fl/fl}$ and Gata3$^{fl/fl\ LckCre}$ mice were infected with 10^4 pfu of HKx31 influenza A virus and BAL fluid was collected at day 10 post-infection. Cells were stimulated with NP or PA peptide for 5 hours and proportion of IFN-**γ**$^+$ and TNF$^+$ cells were assessed by intracellular staining. **(a)** Flow cytometry plots representative of CD8$^+$ T cells stimulated with NP peptide and stained for IFN-**γ**$^+$ and TNF. (**b, c**) Enumeration of the proportion of IFN-**γ** (b) and the proportion of TNF$^+$ cells of the total IFN-**γ**+ population (c). Data are shown as mean ± SEM (*n* = 4 or 5) and are representative of two independent experiments. *$P < 0.05$, **$P < 0.01$, ***$P < 0.001$; .
